# Dystrophinopathies are characterised by impaired cardiac metabolism, contractile dysfunction and fibrosis in patients with and without coxsackie B3 exposure

**DOI:** 10.1186/1532-429X-13-S1-O59

**Published:** 2011-02-02

**Authors:** Joseph Suttie, Sairia Dass, Belen Rial Franco, Rajarshee Banerjee, Pete Cox, Cameron Holloway, Lowri Cochlinl, Alex Pitcher, Jane Francis, Theodoros Karamitsos, Kieran Clarke, Jurgen Schneider, Steffen Petersen, Matthew Robson, Hugh Watkins, Stefan Neubauer

**Affiliations:** 1 Unversity of Oxford, Oxford, UK

## Introduction

Inherited dystrophinopathies (Becker and Duchenne muscular dystrophy, and females with heterozygous mutations) have a high rate of myocardial disease with a variable clinical phenotype. We have previously demonstrated that dystrophinopathic patients have significantly impaired myocardial energetics and fibrosis even in the presence of normal left ventricular ejection fraction. Furthermore, Coxsackie B3 induced viral cardiomyopathy has been shown to be a form of acquired dystrophinopathy.

## Purpose

We therefore acquired 31-Phosphorus magnetic resonance spectroscopy (MRS), proton MRS and tagging to determine the relationship between energetics, lipidosis, fibrosis and regional systolic myocardial strain in patients with inherited dystrophinopathies with and without prior exposure to Coxsackie B3.

## Methods

**Figure 1:**
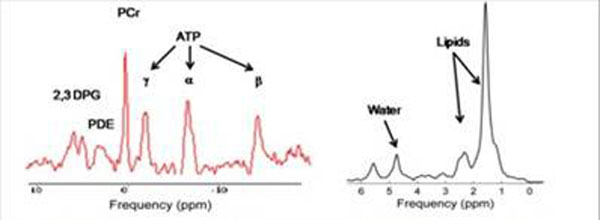
Typical 31-P MRS and proton MRS acquisition in a patient with dystophinopathy

**Figure 2:**
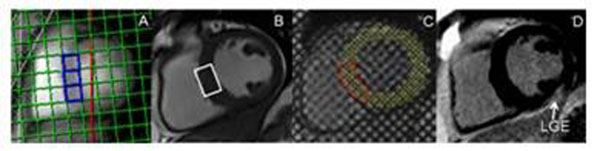
A: 31-Phosphorus energetics spectral voxels; B: Proton spectroscopy voxel; C: Short axis tagging (red border delineates septal region of interst); D: late gadolinium imaging.

Patients (n=22; age 40±12yrs) with dystrophinopathy and preserved ejection fraction (16 males with Becker muscular dystrophy and 6 females with heterozygous mutations; LVEF 65±6%) were scanned using a Siemens Tim Trio 3 T. ELISA IgG assays were performed to determine prior exposure to Coxsackie B. 31P MRS was used to measure the cardiac phosphocreatine to adenosine triphosphate ratio (PCr/ATP) and proton MRS was used to interrogate the myocardial lipid/water ratio (%), both in the mid-ventricular septum (Figure 1 and 2). Results were compared to those from healthy volunteers (n=22). Tagging images (vertical long axis and short axis stack) were acquired to calculate regional strain and torsion (CimTag2D v.7) (Figure 2). Late gadolinium enhancement (LGE) was quantified using QMASS 7.0 software using threshold of 4 standard deviations above unaffected myocardium.

## Results

**Figure 3:**
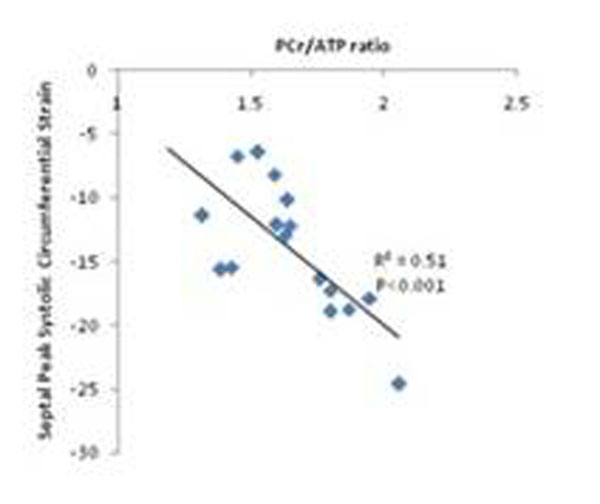
Septal peak systolic circumferential strain vs. phospocreatine to adenosine tri-phosphate (PCr/ATP) ratio in patients with dystronphinopathy.

PCr/ATP ratios were significantly impaired in dystrophinopathic patients (1.6±0.2 vs healthy 2.1±0.3; P=5x10-5). Peak systolic circumferential strain in the mid ventricular septum was impaired in patients with dystrophinopathy (14.1±5.0) compared with healthy controls (18.7±1.8; P=4x10-4) and declined with impaired PCr/ATP ratio (R2=0.51; P<0.001; Figure 3). Proton spectroscopy was performed in the last 11/22 patients and revealed pronounced lipidosis (1.1±0.2% vs. Normals 0.5±0.2%, P<0.001, lipid %=100xlipid/water amplitude). LGE was detected in the lateral wall of 19/22 patients (average 14.7±9%), with no septal fibrosis detected in any patient. Increasing fibrosis did not correlate with impaired energetics, but was significantly higher in positive for Coxsackie B IgG (25±7% vs 10±7%; P=0.001).

## Conclusions

Abnormal myocardial energetics is strongly associated with impaired regional contractility in dystrophinopathies. Myocardial fibrosis, on the other hand, is increased with prior Coxsackie B3 exposure. This provides compelling evidence for a two-hit interaction between inherited and acquired mechanisms of myocardial damage in dystrophinopathies.

